# Cervical cage without plating in management of type II / II A Hangman’s fracture combined with intervertebral disc injury

**DOI:** 10.1186/s12891-015-0734-8

**Published:** 2015-10-06

**Authors:** Fuxin Wei, Le Wang, Zhiyu Zhou, Rui Zhong, Shaoyu Liu, Shangbin Cui, Ximin Pan, Manman Gao

**Affiliations:** Department of Spine Surgery, The First Affiliated Hospital of Sun Yat-sen University, Guangzhou, China; The medical school of Shenzhen University, Shenzhen, Guangdong China; Department of Radiology, The First Affiliated Hospital of Sun Yat-sen University, Guangzhou, China

## Abstract

**Background:**

Surgical intervention is increasingly performed as the primary treatment of unstable Hangman’s fracture. Some authors have advocated using anterior C2/3 discectomy with interbody fusion and plating to treat unstable Hangman’s fracture combined with intervertebral disc injury; however, there are few reports on unstable Hangman’s fracture treated by anterior interbody fusion with the cervical cage (PEEK material) solely.

**Methods:**

This study was to assess the efficacy of the cervical cage in management of unstable Hangman’s fracture combined with intervertebral disc injury. A cohort of 15 patients with unstable Hangman’s fractures fulfilling the inclusion criteria were prospectively submitted to surgical treatment of anterior C2/3 discectomy and interbody fusion using the cervical cage without plating. According to the Levine and Edwards classification, there were 5 type II, and 10 type IIA cases. The clinical outcome (the visual analog scale and the clinical post-traumatic neck score), radiological findings (angulation, translation, and disc height), and bone healing were assessed at 3, 6, 12, and 24 months.

**Results:**

All the patients were followed up successfully. There were no intra- or postoperative complications observed. Solid fusion was achieved in all cases by 6 months after surgery. The local kyphotic angle was corrected significantly with the mean preoperative 12.31 ± 2.96 degrees, initial postoperative −1.98 ± 1.62 degrees and the latest follow-up −1.72 ± 1.60 degrees respectively (*P* < 0.05).The translation was also corrected significantly with the mean preoperative 3.20 ± 1.16 mm, initial postoperative 0.97 ± 0.36 mm, and the latest follow-up 1.05 ± 0.34 mm respectively (*P* < 0.05). The mean visual analog scale and the clinical post-traumatic neck score improved significantly following surgery (*P* < 0.05).

**Conclusions:**

This case series demonstrates that anterior C2/3 discectomy and interbody fusion with the cervical cage solely is effective and reliable in management of type II / IIA Hangman’s fracture with C2/3 disc injury when properly indicated.

## Background

Hangman’s fracture, or traumatic spondylolisthesis, which accounts for 4–7 % of all cervical fractures/dislocations [1], is the second most common fracture of the second cervical vertebra [2]. It involves a bilateral arch fracture of C2 with a variable degree of displacement of C2 corpus on C3 vertebrae. Opinions vary regarding the optimal treatment of Hangman’s fracture. Although most Hangman’s fractures are managed conservatively [3, 4], the optimal strategy remains controversial, especially for the type II and II A injuries (according to the classification of Levine and Edwards [5]) which are thought to be unstable.

Most authors recommended that surgical intervention should be reserved for cases with failure of conservative measures; however, pseudoarthrosis, anterior dislocation, angulation of C2 over C3, recurrent axial pain were observed in about 60 % of the cases of type II / II A injuries that were primarily treated with conservative therapy, suggesting the need for an early operation in patients with unstable Hangman’s fractures [3, 6–8]. In addition, conservative treatment has several disadvantages, such as prolonged immobilization with cervical tong traction and/or the halo device for an average of 3–6 months with an uncertain outcome. In particular, in elderly patients, prolonged immobilization might be intolerable. Therefore, the authors preferred early surgical treatment of unstable Hangman’s fracture.

Both anterior and posterior approaches can be used to treat lesions at C2; however, the optimum surgical treatment is controversial [9]. The posterior approach, by which we can avoid major visceral and vascular structures, was preferred for its simple exposure, however, inserting transpedicular screws poses the risk of intraoperative neurological and vascular injuries, which was reported from 11–66 % of injury rate in its early application [10–12]. The anterior approach, which has the advantage of technical ease and a relatively short fusion, is characterized by C2-3 discectomy and interbody fusion with plating [13]. Some authors have reported good clinical results of this approach in management of Hangman’s fractures, especially for the patients with C2/3 intervertebral disc injury, however, there are few reports on unstable Hangman’s fracture treated by anterior discectomy and interbody fusion (ACDF) using cervical cage without plating, which could shorten the duration of surgery and medical cost, especially for those combined with C2/3 disc injury and disease of vertebral body, such as bone cyst, which could not be inserted screws with plating.

Is anterior C2-3 discectomy and interbody fusion with the cervical cage solely effective and reliable in management of unstable Hangman’s fracture combined with C2/3 intervertebral disc injury? To answer the question, we designed this prospective study to assess the clinical and radiologic outcomes of this innovative technique.

## Methods

This study was approved by the institutional review board of the first affiliated hospital of Sun Yat-sen University. Written informed consent had been provided by the subjects (or their representatives) involved in this study. From December 2009 to March 2013, 15 patients with Hangman’s fractures were included in this study. The inclusion criterion required the following: type II, IIA (according to the classification of Levine and Edwards [5]) fracture combined with C2/3 disc injury with or without neurologic impairment and age of 20–60 years (nonpathologic adult). Patients with unstable vital signs, severe internal disease or skull fractures, pathological fractures, or cervical vertebral body fractures were excluded.

This study included 10 males and 5 females with an average age of 36.8 years (range, 26–58 years) at the time of operation. Clinical information was shown in Table 1. Referring to the Levine-Edwards classification, 10 patients (66.7 %) were in type IIA and 5 patients (33.3 %) were in type II. Axial pain and restricted motion of the spine were complained from all patients immediately after injury. According to the American Spinal Injury Association scale [14], 2 cases were graded as spinal injury C, 4 cases were D, and grade E for other cases.

**Table 1:** Summary of the Data of 15 Patients in This Study

Case	Age/gender	Injury	Diagnosis	ASIA	Hosp.stay (days)	Results
1	30/F	VA	II + STI + fracture of right radius	C	12	Union
2	30/M	VA	IIA + STI	E	7	Union
3	38/M	VA	IIA	D	6	Union
4	25/F	VA	IIA + STI	E	7	Union
5	35/M	Others	IIA + HI	D	9	Union
6	40/M	VA	IIA + STI	E	7	Union
7	45/F	VA	II + STI	E	5	Union
8	59/F	Falling	IIA + HI + STI	D	7	Union
9	30/M	VA	II + fracture of left humerus	D	7	Union
10	40/M	Others	IIA + HI	E	6	Union
11	26/F	VA	II + STI	E	5	Union
12	28/M	VA	IIA + HI + STI	E	6	Union
13	58/M	Falling	II + fracture of right raidus	E	7	Union
14	32/M	VA	IIA + STI	C	14	Union
15	29/M	VA	IIA + STI	E	5	Union

*VA* indicates vehicle accident, *STI* soft tissue injury; *HI* head injury; Others, hit by a heavy falling objects, *ASIA* American Spinal Injury Association, *FU* follow-up

Skull traction was performed for all the cases preoperatively. According to the type of the individual case, a weight of 3–5 kilogram with an appropriate angle was applied to stabilize and reduce the fracture. At least 50 % degree of reducing was accomplished in all the cases without advanced neurological deficits or deterioration. All patients performed neck extension exercises under constant traction without neurologic deterioration.

Anterior C2/3 cervical discectomy and autologous bone fusion with a polyetherether ketone (PEEK) cage (Solis, Stryker Corporation, Cestas, France) was performed in each patient by the same senior author. The patient was placed in the supine position with the neck slightly extended and 3–5 kg of axial traction. After anesthesia, fiberoptic bronchoscope-guided nasal intubation was performed. The head of the patient was taped and turned away from side of incision. Surgical procedure was performed using a standard anterior Smith-Robonson approach. A longitudinal incision was made from the angle of the jaw to the hyoid bone. After C2/3 anterior exposure was obtained, all the patients were found to have a partial tear of the anterior longitudinal ligaments and disc disruption. To facilitate decompression, stable placement of self-retaining retractors were used in operation. Retraction on the cephalad of the incision was best accomplished by bending a malleable retractor, which could be used as a lever against the vertebral body of C2. After elevating the soft tissues cephalad and superiorly, we got enough exposure to access the disc level and to place instruments. It was helpful to avoid injury of the esophagus and sympathetic chain by careful placement of retractors. Microscope-assisted discectomy and decompression were performed followed by removing the skull traction after distracting the disc space using the casper system under fluoroscopic guidance. The endplates were curetted to remove cartilage and the bony endplates were preserved. Under continuous imaging control, the fracture reduction was reduced as much as possible by placing the head in a more slightly extended position and pushing the vertebral body of C2 backward gently to close the remaining gap. After that, the iliac-crest bone graft was harvested. We used a specialized hollow cylindrical gouge that accompanied the Solis instrumentation set to core a critical amount of cancellous bone out from the iliac crest between the inner and outer tables. Intraoperative sizing was performed for the cage using the templates under fluoroscopic guidance. The cage was filled with comminuted bone graft and tightly impacted into the prepared disc space. Finally, the reduction and proper position of the cage was ascertained again under imaging control.

Closure of the wound was performed in layers with the routine use of a suction drain which was removed within 24 hours. All patients had prophylactic antibiotic coverage for 24 hours. Postoperative immobilization was accomplished with a hard cervical collar for 6–10 weeks.

**Fig. 1:**
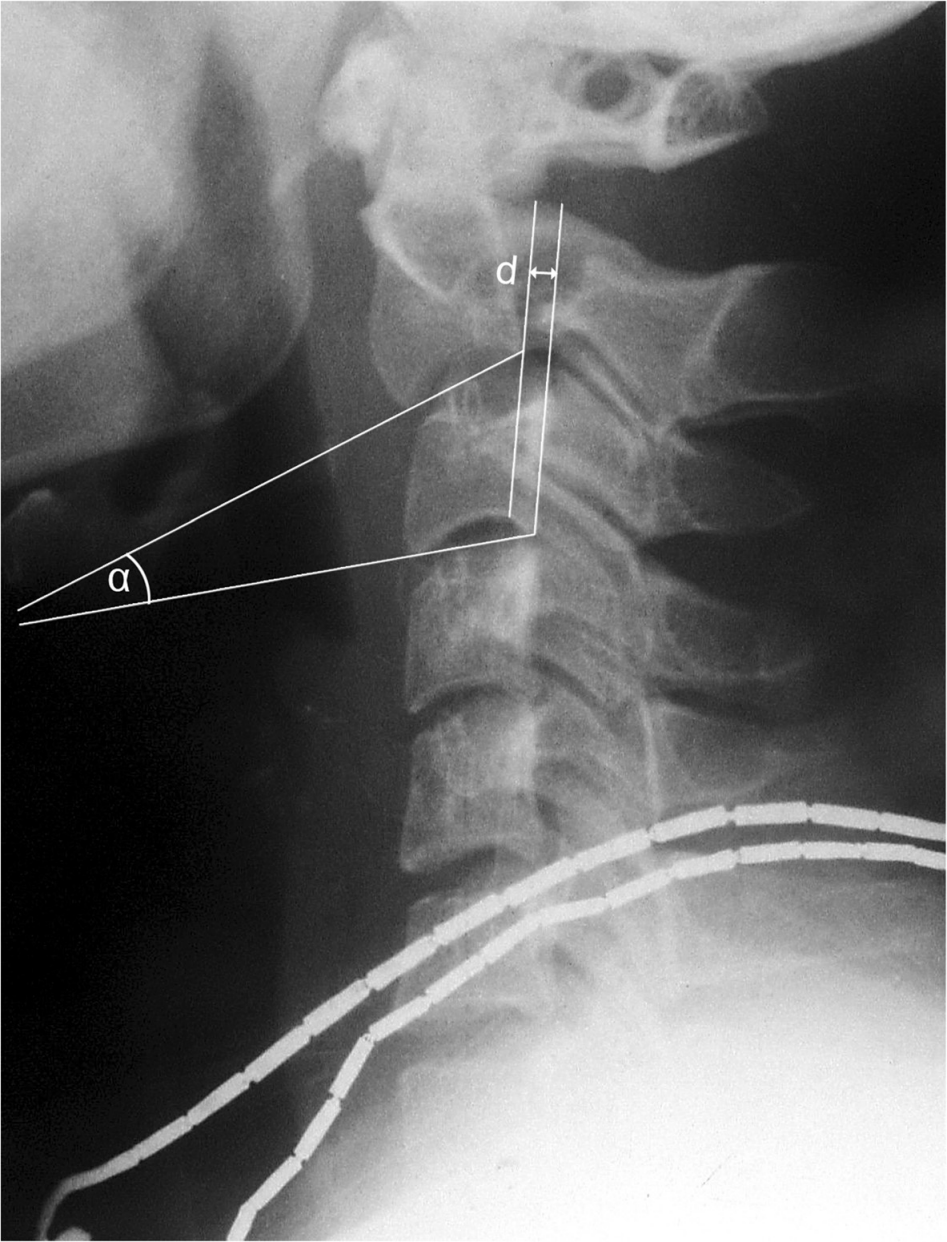
Diagram showing the local kyphotic angle and translation. *α* is the angle between inferior border of C2 and s C3. *d* is the distance between posterior boarders of C2 and C3

Patients were followed up at 3, 6, 12 and 24 months after operation. Clinical and radiological outcomes were assessed respectively. Clinical assessment was performed by an independent examiner at each visit, using the visual analog scale (VAS) form for neck pain and the clinical post-traumatic neck score (PTNC) [15] which contains critical information such as cervical movement, neurological statue and daily leaving activities. Radiological assessment involved plain x-ray film assessment of local kyphotic angle of C2/3, the anterior translation of C2 pre and postoperatively, and the postoperative disc height changes of C2-3. The local kyphotic angle was defined as the angle formed by lines drawn along the inferior endplate of axis and the inferior endplate of C3 (Fig. 1) [16]. Translation was measured as the distance between parallel lines drawn through the posterior border of C3 and the inferior endplate of C2 (Fig. 1) [17]. The disc height was the mean of the sum of the vertical distance between the anterior and posterior edges of the vertebral end plates. [17]

At 3 and 6-month follow-up, dynamic lateral flexion/extension radiographs and additional CT scans were also performed for all the patients to ascertain the fusion status. The criteria of fusion were as follows [18]: (1) trabecular bone across the interfaces and connects superior and inferior vertebral bodies; (2) radiolucency inside the cage disappeared; (3) Adequate disc height was restored, without collapse-induced kyphosis; (4) the flexion-extension range of motion at the fusion site was 2° or less.

All the images were reviewed by the same radiologist and assessor who were blinded to the clinical outcome of the patients. Measurements were done on digital radiographs with in-built software to measure distance and angle up to the accuracy of 0.01 mm and 0.1 degree respectively (Philips DICOM Viewer R2.5, Philips Medical Systems Nederland B.V., Best, The Netherlands).

The SPSS (version16.0, Chicago, IL, USA) package was used for the statistical analysis. The data of the ASIA scale, VAS, PTNC score and local kyphotic angle were analyzed using the Wilcoxon signed ranks test, with a confidence interval of 95 %. The data of translation were analyzed using One-way Analysis of Variance. The data of disc height between initial postoperative and the latest follow-up was compared by using paired t test. Data were presented as the mean ± standard deviation. Statistical significance was indicated at *P* < 0.05.

## Results

**Fig. 2:**
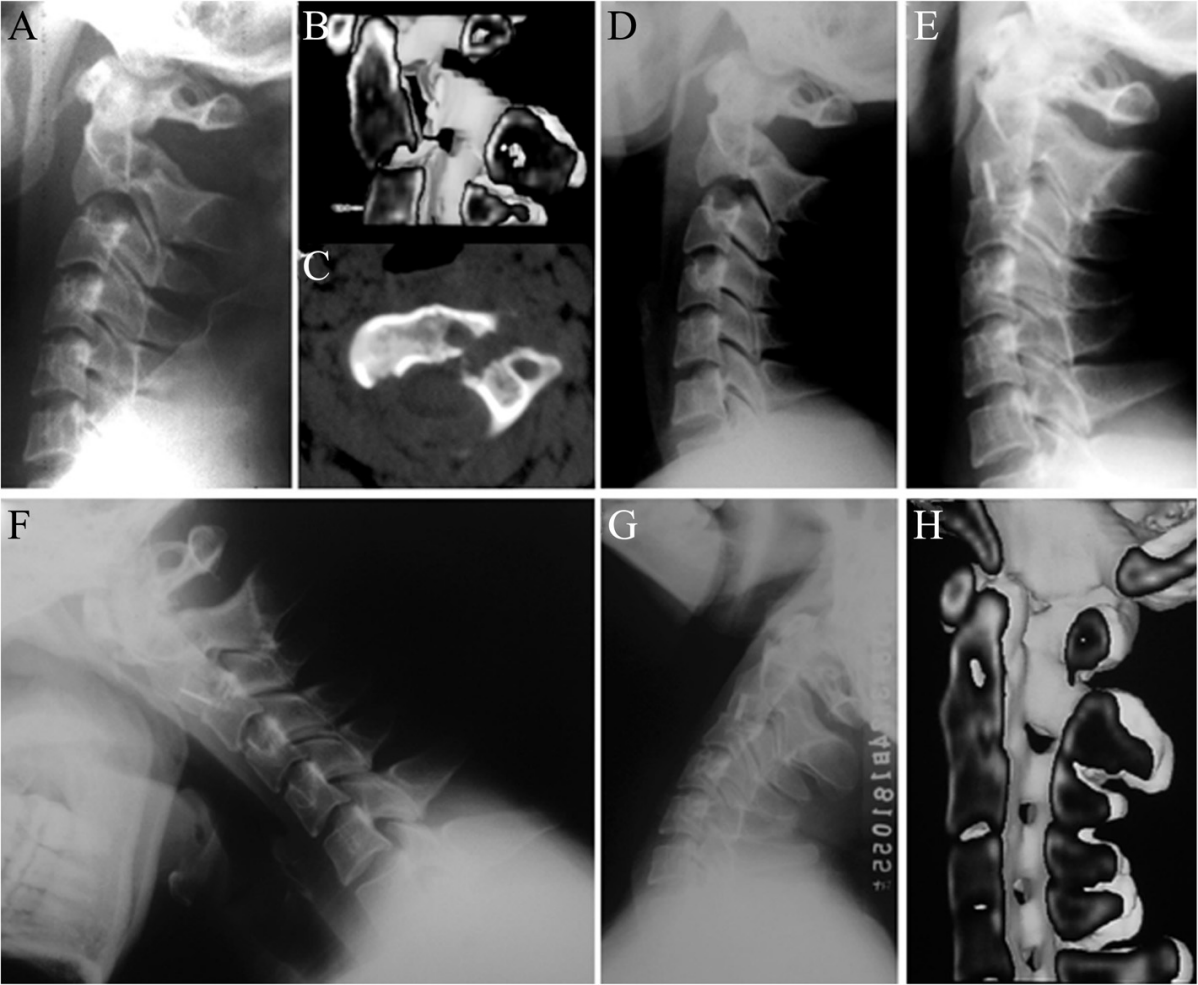
Images of a 38-year-old male patient. **a** and **b**, preoperative lateral X-ray and CT scans showing a type IIA hangman’s fracture with severe angulation. **c**, CT with axial section showing a bone cyst in the vertebral body of C2. **d**, Some degree of reducing was accomplished during skull traction for 3 days. **e**, 3-month postoperative lateral X-ray showing adequate reduction and bony fusion. **f** and **g**, 24-month flexion/extension lateral X-rays showing no range of motion at the fusion site. **h**, CT with sagittal reconstruction showing solid fusion and fracture healing

**Fig. 3:**
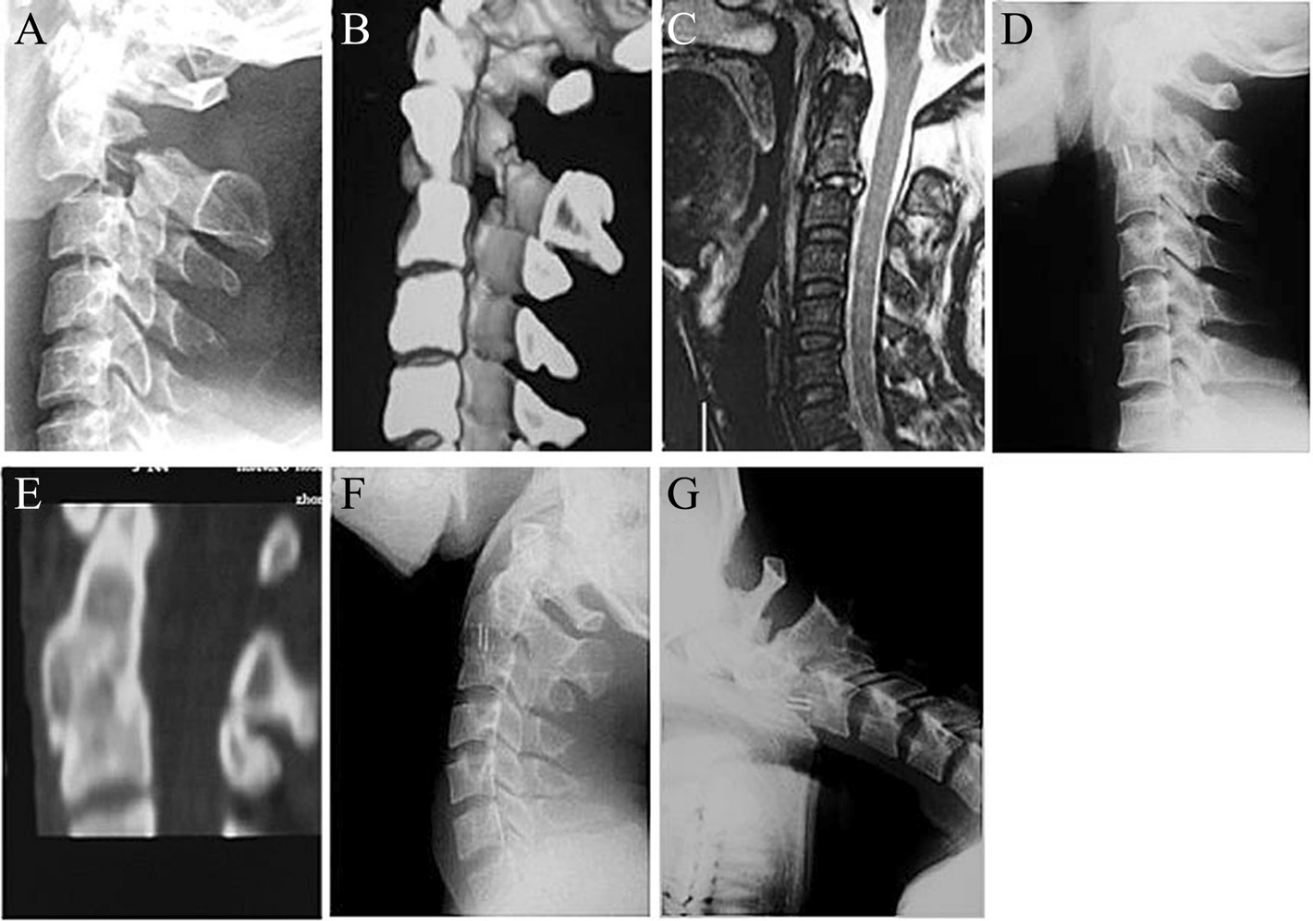
Images of a 40-year-old male patient. **a** and **b**, preoperative lateral X-ray and CT scans showing a type II Hangman’s fracture with severe translation. **c**, MRI with sagittal section showing C2/3 intervertebral disc injury. **d**, 3-month postoperative lateral X-ray showing adequate reduction and bony fusion. **e**, CT with sagittal reconstruction showing solid fusion and fracture healing. **f** and **g**, 24-month flexion/extension lateral X-rays showing no range of motion at the fusion site

The average total operative time was 93.5 minutes (range 82–130 minutes) with blood loss was 23.3 cc (range 20–50 cc). The average hospital stay was 7.3 days (range4-15 days). All the patients were followed up successfully. Fusion was evident at 3–6 months postoperatively in all cases (Figs. 2 and 3).

A summary of clinical outcomes was provided in Table 2. Of the total 15 patients, there was a significant improvement between preoperative VAS and either 3-month follow up or 24-month follow-up VAS score (z = −3.431, *P* < 0.01; z = −3.436, *P* < 0.01, respectively, Table 3). Significant improvement was also achieved between preoperative PTNC and either 3-month follow-up or the 24-month follow-up PTNC (z = −3.409, *P* < 0.01; z = −3.414, *P* < 0.01, respectively, Table 3).

**Table 2:** Summary of VAS and PTNC scores

	VAS Score			PTNC Score		
Case No.	Preop	3-month FU	12-month FU	Preop	3-month FU	12-month FU
1	9	2	1	28	76	95
2	5	0	0	53	78	95
3	7	0	0	35	81	98
4	6	1	0	55	68	96
5	7	1	0	53	79	98
6	8	3	1	21	58	93
7	6	0	0	35	81	96
8	8	1	1	31	76	93
9	8	1	0	35	73	98
10	5	1	0	56	68	95
11	7	1	0	51	76	93
12	3	0	0	59	78	100
13	8	2	2	41	73	93
14	4	2	0	58	76	100
15	7	1	0	35	68	98

VAS indicates visual analog scale; PTNC, post-traumatic neck score; FU, follow up

**Table 3:** Clinical and Radiological Outcomes Pre and Postoperatively

	VAS	PTNC	Local Kyphotic Angle (°)	Translation (mm)
Preoperative	6.5 ± 1.7	43.1 ± 12.5	12.31 ± 2.96	3.20 ± 1.16
Postoperative	1.1 ± 0.9	73.9 ± 6.2	−1.98 ± 1.62	0.97 ± 0.36
Follow up	0.3 ± 0.6	96.1 ± 2.5	−1.72 ± 1.60	1.05 ± 0.34
P*	0.006	0.004	0.001	0.001
P**	0.010	0.033	0.681	0.131

All values are shown as average ± standard deviation

*VAS* indicates visual analog scale, *PTNC* post-traumatic neck score

P*: Postoperative VS Preoperative

P**: Follow up VS Postoperative

Neurological status improved from C and D to E in all 6 cases. No patient experienced worsening neurological function postoperatively. There was significant postoperative neurologic improvement compared with patients’ preoperative neurologic function (z = −2.136, *P* = 0.003).

A summary of radiological outcomes is provided in Table 4. The preoperative and initial postoperative local kyphotic mean angle was 12.31 ± 1.8° and −1.98 ± 1.62° respectively, which differed significantly (*P* < 0.01, Table 3). The mean translation of C2 also improved significantly after surgery (z = −3.408, *P* < 0.01, Table 3). There were no significant correction loss of radiological results at the final follow up (z = −1.710, P > 0.05, Table 3). The initial postoperative and the latest follow-up mean C2-3 disc height was 7.10 ± 0.74 mm and 7.07 ± 0.73 mm respectively, which did not differ significantly (t = 1.970, *P* = 0.069).

**Table 4:** Summary of Radiological Measurements Pre and Postoperatively

	Local kyphotic angle (°)			Translation (mm)		
Case No.	Preop	Initial Postop	The Latest FU	Preop	Initial Postop	The Latest FU
1	13.2	−1.8	−1.3	4.88	1.23	1.21
2	12.5	−1.2	−1.1	2.40	0.86	1.05
3	14.8	−2.6	−2.4	2.21	1.15	0.94
4	13.3	−1.1	−0.3	2.56	0.63	0.52
5	13.9	−1.4	−1	2.13	0.59	0.46
6	13.1	−4.2	−4.6	2.37	0.54	1.04
7	6.6	−1.9	−2	4.64	0.62	0.89
8	13.5	−3.6	−2.9	1.75	1.01	1.1
9	9.2	−0.8	−0.8	4.93	0.73	0.7
10	12.1	−3.9	−3.6	2.82	0.58	0.81
11	16.8	1.1	1	5.47	0.52	0.67
12	12.3	−2.6	−2.5	2.91	1.28	1.29
13	7.2	1	1.2	3.84	0.77	1.02
14	12.5	−2.3	−1.7	2.25	1.65	1.63
15	13.6	−4.4	−3.8	1.78	1.33	1.4

FU indicates follow up

**Fig. 4:**
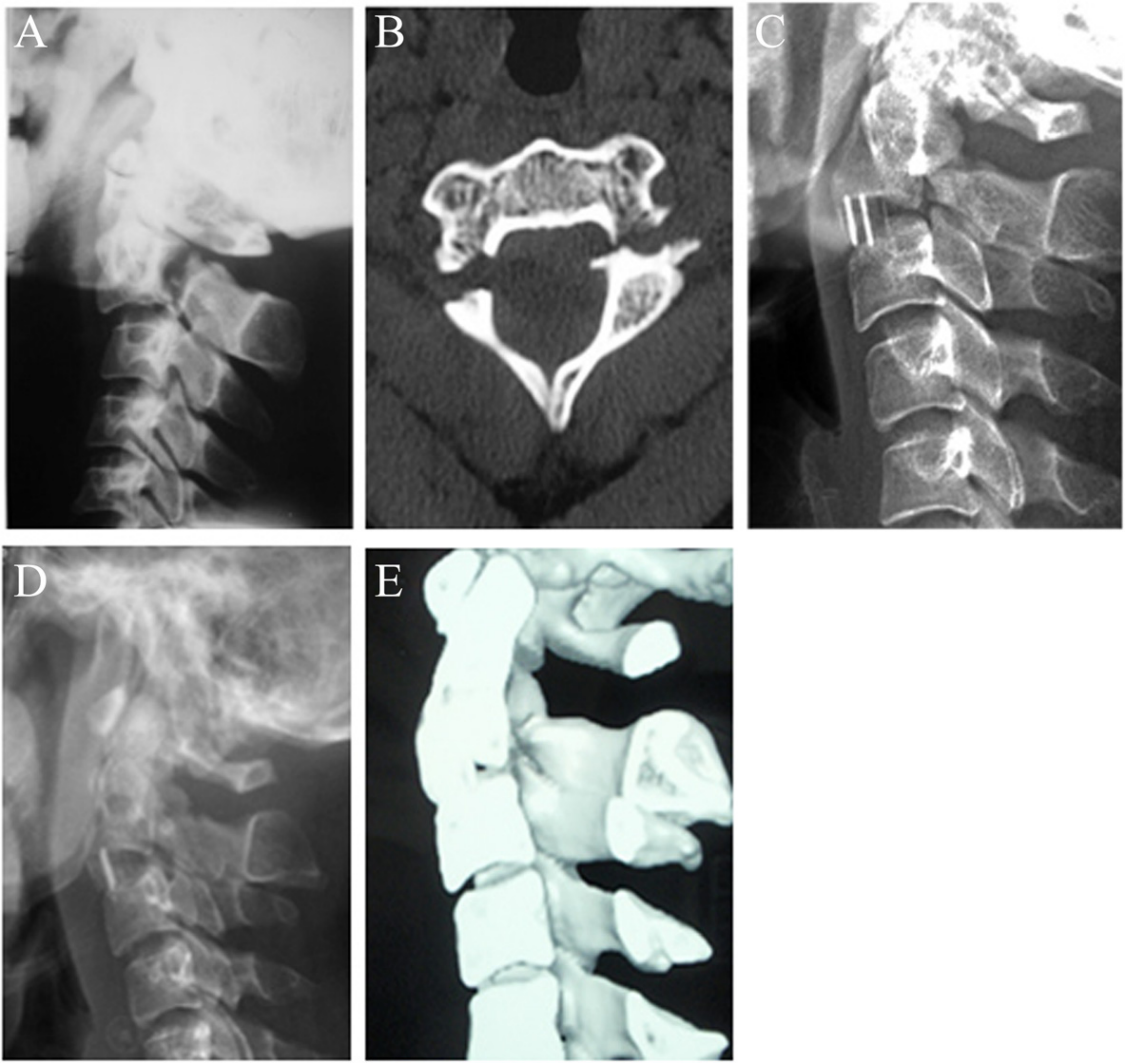
Images of a 26-year-old female patient. **a** and **b**, preoperative lateral X-ray and CT scans showing a type II hangman’s fracture with severe translation. **c**, Postoperative lateral X-ray showing minor implant migration at 1 week postoperatively. **d**, The X-ray showed the implant remained the same position during follow-up. **e**, Bone fusion was confirmed using CT scan at 4 months postoperatively

The complications observed included minor implant migration of one patient at 1 week postoperatively without any symptom complained. Cervical orthosis was administrated to limit movement after being discharged. The X-ray showed the implant remained the same position during follow-up. Bone fusion was confirmed using CT scan at 4 months postoperatively (Fig. 4). One case suffered superficial infection, which was cured 2 weeks after medication.

## Discussion

Various surgical or nonsurgical treatments of Hangman’s fracture have been described, but the optimal treatment remains in question [3, 4, 19]. Although nonsurgical treatments were widely favored in the primary management of a Hangman’s fracture, healing was slow and uncertain, and the course of treatment for the fracture was long. Studies have shown that anterior dislocation, angulation of C2 over C3, pseudarthrosis, and recurrent axial pain occurred in about 60 % of patients with type II, IIA and III fractures after conservative treatment [3, 5, 19]. It was reported that the union rates following conservative management in type II, IIA and III fractures were 60, 45, 35 % respectively [9]. Some authors insisted that nonsurgical management was inappropriate in patients with unstable Hangman’s fractures and discoligamentous injuries, due to the absence of a blood supply to the disc, which is unable to repair itself. [20] This frustrating fact could explain why many surgeons choose primary operation in management of unstable Hangman’s fracture [8, 21–23], which could shorten the course of treatment [24]. Another reason for operation is that surgical intervention is employed in the hope of improving neurological outcome [5] or for patients suffering persistent pain from the cervical spine after treated with an external orthosis.

Surgical stabilization has been accomplished in both anterior and posterior approaches. Due to the complex anatomic feature of the upper cervical spine, the posterior approach was preferred for its relative simple exposure with no major vascular or visceral structure. Among the different posterior approaches, direct posterior fixation of the pedicles or pars fracture with a screw across the fracture line was reported with the advantage of motion reservation in C2-C3 [7, 21, 25]. However, it had been reported that it was ineffective in patients with unstable fractures due to discoligamentous injury in C2-C3, failing of preventing kyphosis and loss of disc height. [26] Redislocations in discoligamentous unstable Hangman’s fracture following direct pars repair have also been reported [4, 26]. Although pedicle screw fixation has been reported with good clinical outcomes, it posed the risks of intraoperative neurological and vascular injuries [10–12]. Yukawa et al. [27] reported the perforation rate of pedicle screws in C2 and C3 was 21.6 %. Although a computer-guided surgical navigation system has been carried out to improve the accuracy of screw insertion [8], these systems were not installed in most hospitals owing to their high cost and user unfriendliness. Another shortcoming of posterior approach was the axial pain after operation.

In this instance, we advocated an anterior C2-3 discectomy and fusion (ACDF) for unstable hangman’s fracture, which has been confirmed to be an effective strategy [3, 23, 28]. Although a high anterior exposure was once considered to be complex and might impose a risk to vital structures, all the incisions were exposed without difficulty and there were no intraoperative complications reported in this study. In our series, the standard anterior Smith-Robonson approach was used, which was same as what Tuite et al. described in 1992 [6]. According to our experience in the 15 cases, anterior approach offered satisfied exposure for reduction and arthrodesis. Pre and intraoperative axial traction with slightly neck extension of the patients combined with the head turning away from side of incision facilitate the exposure. In extension, the mandible was more cephalic, which could save more space for the cephalic movement of the retractor, then facilitate the stretching range of exposure.

Many of the ACDF-associated complications were graft-related problems. [29, 30] Cervical anterior plate systems were introduced in response to some of these problems; however, plate-assisted fusion has itself been associated with plate fracture, screw back-out and fracture, and soft-tissue injury to structures such as the esophagus [31, 32]. Although the utilization of anterior cervical plates helps to shorten the duration of postoperative immobilization, it increases the duration of surgery and is associated with problems of soft-tissue injury as well as instrumentation failure [33]. In particular, anterior cervical plating is inappropriate for patients with poor bone quality of cervical vertebral body, such as bone cyst, which could not provide sufficient pullout strength at the screw-bone interface. Interestingly, Savolainen et al. prospectively compared the fusion rate of autologous bone graft with or without plus plating on anterior operations for cervical spondylosis. Of these, both of the two groups achieved 100 % fusion rate [34].

In this study, with the advent of minimal invasive surgery, we utilized the cervical cage (PEEK material) without plating for C2-3 discectomy and fusion (ACDF) in management of type II / IIA Hangman’s fractures, which required a less invasive approach and showed good clinical and radiological results at the latest follow-up (Figs. 2 and 3). A concern arises that it might not provide adequate stability for the Hangman’s fracture. The configuration of the superior and inferior surfaces of the cage conforms to the shape of the respective opposing surface of the disc space, and it has retention teeth as well as bilateral titanium spikes on the superior and inferior surfaces, which could provide a secure fixation and prevent migration/extrusion of the cage. Furthermore, the cage was made of PEEK material, a thermoplastic material with high molecular weight, whose elasticity modulus was similar to that of bone [33]. This helped to minimize stress shielding and subsidence of the cage and allowed optimum interaction of compressive forces at the graft-host interface, which could avoid significant subsidence and result in high fusion rate that our study has confirmed. We also performed biomechanical evaluation of this cage for type II Hangman’s fracture, which showed that there were no significant difference in range of motion (ROM) of lateral bending, rotation and extension between the cage group and bone graft plus plating group, except ROM of flexion, which could be partly compensated by hard cervical collar [35]. However, it is imperative to emphasize that this surgical method can not be applied to all the Hangman’s fracture cases.

The fact that one patient in this study manifested evident minor implant migration was also given close attention. In this case, the local kyphotic angle was more than 15° combined with more than 5.0 mm translation of C2 preoperatively. Therefore, in our late clinical practice, candidates for this study were strictly limited to the patients with local kyphotic angle of 15° or less and translation of 5.0 mm or less. Since then, in our follow up, we have not seen any case with implant migration yet. One important reminder is that we did not include type III fractures in our study due to low incidence. We suggest not to utilize this surgical method to treat type III Hangman’s fracture due to severe instability.

The incidence of donor site morbidity has been reported to be as high as 20–30 % in some series of ACDF, and deficits included acute and chronic pain, infection, and nerve injury [29, 30, 36]. Harvesting of structural corticocancellous autologous bone from the iliac crest may lead to excessive pain and morbidity at the donor site, as well as iliac crest fracture [37, 38]. In our series, because there was no need for a structural graft, cancellous bone was harvested via a much smaller opening than with earlier methods, reducing the incidence of morbidity. There were no complications related to the donor site in this study. Several authors have reported the placement of cages filled with bone substitute or even those that are empty; [39, 40] however, there is evidence, that the patient’s own bone is associated with the best fusion rates [41, 42].

Following a Hangman’s fracture, lots of patients suffer chronic neck pain sufficiently intense to affect daily life. Some authors ascribed the axial neck pain to the cervical disc injury or to the presence of a fracture on the inferior facet of the axis [43]. Another reason of residual neck pain may be local kyphosis at C2-3 [43], as well as the absence of fusion or pseudarthrosis after opration. In this study, no patient complained of suffering neck pain at the latest follow up. This could be contributed to C2/3 discectomy and solid fusion. However, this needed to be verified by further study and the results of a long-term follow up.

The hospitalization period was relatively short (4–15 days with an average of 7.3 days) and the need for longer hospitalization was attributed to the patients with head trauma and fractures of extremities, who required the help of the nursing and physical therapy staff, allowing early mobility of the patients, their removal from the bed, and the reduction of the lesions.

The authors appreciated the limitations of any stand-alone device when compared with an anterior plate. Postoperative external immobilization is certainly necessary in the absence of a plate; however, the risk of device extrusion was probably less with this kind of cervical cage as a s result of titanium pins and retention teeth over the superior and inferior surfaces.

In our study, the anterior approach was especially appropriate for Hangman’s fracture combined with intervertebral disc injury or disc herniation compressing the spinal cord. Using the anterior approach with this kind of cervical cage in management of these fractures, solid fusion was achieved in all cases without complications. In our experience, the anterior approach with the cervical cage stabilization without plating, which can shorten the duration of surgery and medical cost, may be an alternative for type II / IIA Hangman’s fracture when properly indicated.

However, the current case series have some limitations. It was a small-sized prospective study and the number of patients was restricted due to the low incidence of Hangman’s fracture. Another limitation is that this study was just a preliminary report about an early technical experience based on the results of 2-year’s follow up. A multicenter prospective controlled study of Hangman’s fracture should be considered in the future.

## Conclusions

This study demonstrates that anterior C2/3 discectomy and interbody fusion with the cervical cage solely is effective and reliable in management of type II / IIA Hangman’s fracture with C2/3 disc injury when properly indicated. One important reminder is that this method is not suitable for the treatment of all Hangman’s fracture cases. Further studies are warranted to clarify its proper indication.

## Abbreviations

ACDF: Anterior discectomy and interbody fusion
PEEK: Polyetherether ketone
VAS: Visual analog scale
PTNC: Post-traumatic neck score
ROM: Range of motion
